# Supplementary material for: Molnupiravir (MK-4482) is efficacious against Omicron and other SARS-CoV-2 variants in the Syrian hamster COVID-19 model
Source: bioRxiv. 2022 Feb 23:2022.02.22.481491. Preprint. [Version 1] doi: 10.1101/2022.02.22.481491 (PMC8887072; doi:10.1101/2022.02.22.481491)
Supplement: 1 [file NIHPP2022.02.22.481491V1-supplement-1.pdf]

bioRxiv preprint doi: https://doi.org/10.1101/2022.02.22.481491; this version posted February 24, 2022. The copyright holder for this preprint (which was not certified by peer review) is the author/funder. This article is a US Government work. It is not subject to copyright under 17 USC 105 and is also made available for use under a CC0 license.

SUPPLEMENTARY MATERIAL

Title: Molnupiravir (MK-4482) is efficacious against Omicron and other SARS-CoV-2 variants in the Syrian hamster COVID-19 model

Authors: Kyle Rosenke[1], Atsushi Okumura[1], Matthew C. Lewis[1], Friederike Feldmann[2], Kimberly Meade-White[1], W. Forrest Bohler[1], Amanda Griffin[1], Rebecca Rosenke[2], Carl Shaia[2], Michael A. Jarvis[1,3,4]*, Heinz Feldmann[1]*

Affiliations: [1]Laboratory of Virology, [2]Rocky Mountain Veterinary Branch, National Institute of Allergy and Infectious Diseases, National Institutes of Health; Hamilton, MT, USA.

[3]School of Biomedical Sciences, University of Plymouth; Plymouth, Devon, UK.

[4]The Vaccine Group Ltd; Plymouth, Devon, UK.

***Corresponding authors:**

Heinz Feldmann. Email: feldmannh@niaid.nih.gov

Michael A. Jarvis. Email: michael.jarvis@plymouth.ac.uk

bioRxiv preprint doi: https://doi.org/10.1101/2022.02.22.481491; this version posted February 24, 2022. The copyright holder for this preprint (which was not certified by peer review) is the author/funder. This article is a US Government work. It is not subject to copyright under 17 USC 105 and is also made available for use under a CC0 license.

SUPPLEMENTARY FIGURE 1

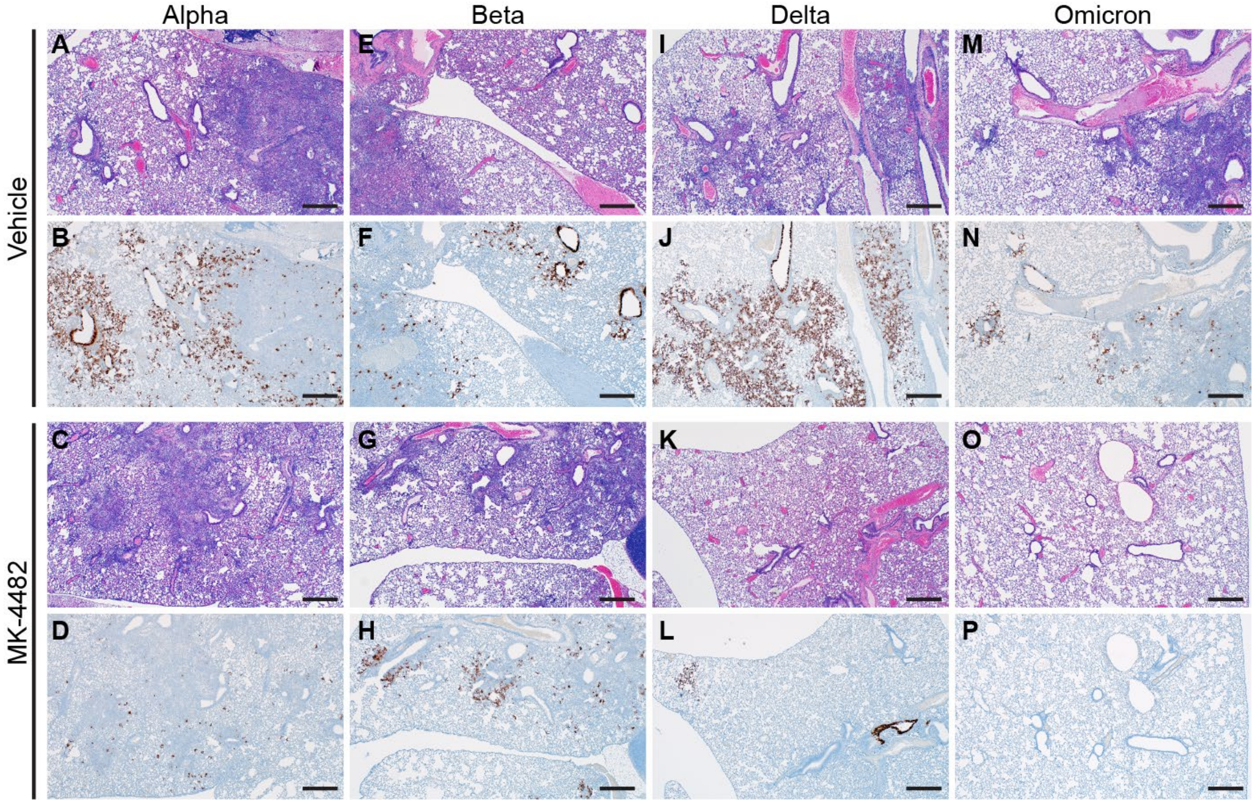

Fig. S1. MK-4482 efficacy on lung pathology in hamsters infected with multiple SARS-CoV-2 VOCs. The experimental design is shown in Figure 1A. Lung tissue was collected on 4 dpi and prepared for hematoxylin and eosin (H&E) stain and immunohistochemistry (IHC) using an antibody directed against the SARS-CoV-2 nucleocapsid protein. **(A, E, I, M) Lung H&E (40x).** H&E stain from vehicle treated hamsters infected with Alpha, Beta, Delta and Omicron VOC, respectively, showing bronchointerstitial pneumonia and vasculitis. **(B, F, J, N) Lung IHC (40x).** SARS-CoV-2 nucleoprotein detection in lung section from vehicle-treated hamsters infected with Alpha, Beta, Delta and Omicron, respectively, exhibiting immunoreactivity associated with areas of pneumonia (brown color). **(C, G, K, O) Lung H&E (40x).** H&E stain from MK-4482 treated hamsters infected with Alpha, Beta, Delta and Omicron VOC, respectively, showing reduced bronchointerstitial pneumonia. **(D, H, L, P) Lung IHC (40x).**

bioRxiv preprint doi: https://doi.org/10.1101/2022.02.22.481491; this version posted February 24, 2022. The copyright holder for this preprint (which was not certified by peer review) is the author/funder. This article is a US Government work. It is not subject to copyright under 17 USC 105 and is also made available for use under a CC0 license.

SARS-CoV-2 nucleoprotein detection in lung section from MK-4482 treated hamsters infected with Alpha, Beta, Delta and Omicron VOC, respectively, exhibiting reduced or no immunoreactivity. Each panel shows a lung section from a representative hamster. Bar = 500 µm

SUPPLEMENTARY FIGURE 2

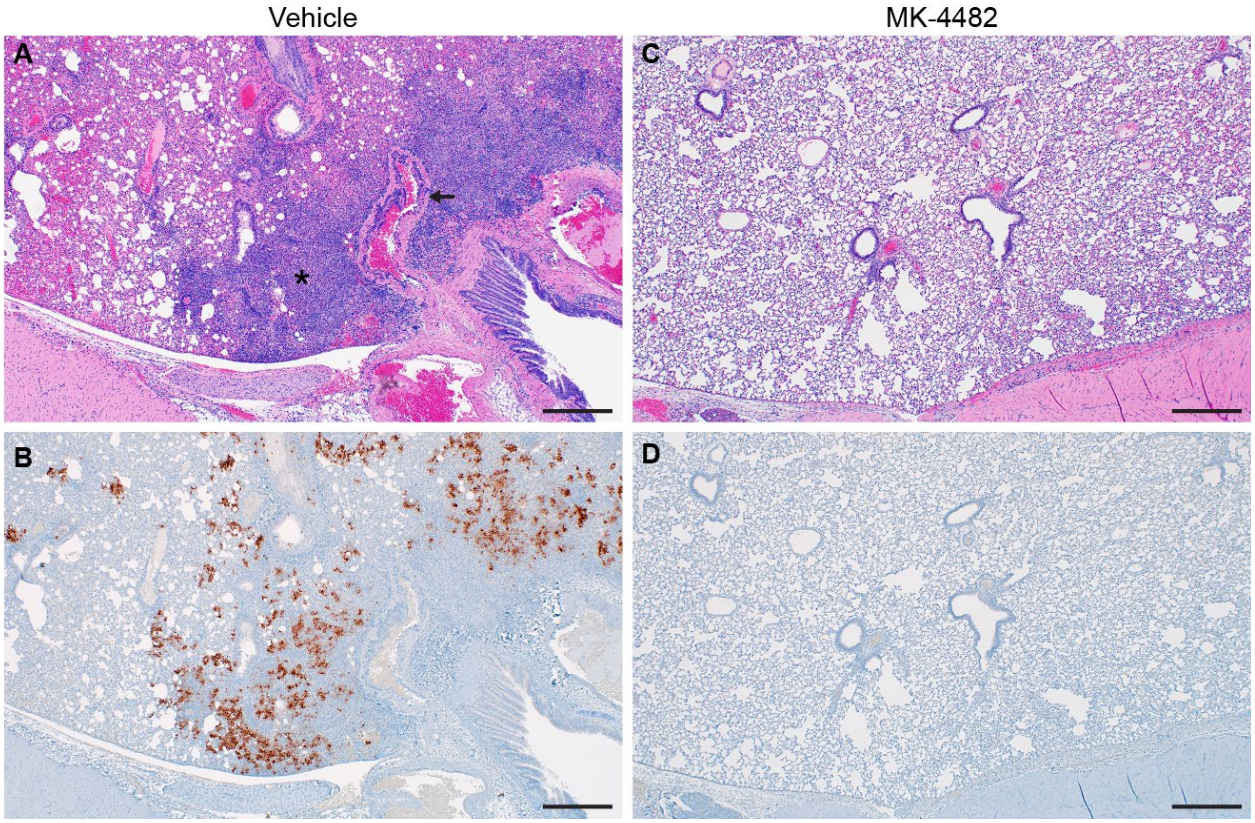

Fig. S2: MK-4482 efficacy on lung pathology in hamsters infected with a high dose of Omicron SARS-CoV-2 VOC. The experimental design is shown in Figure 1A. Lung tissue was collected on 4 dpi and prepared for H&E stain and IHC using an antibody directed against the SARS-CoV-2 nucleocapsid protein. **(A) Lung H&E (40x).** Lung H&E of a vehicle treated hamster infected with Omicron exhibiting a focus of bronchointerstitial pneumonia (asterisk) and vasculitis (arrow). **(B) Lung IHC (40x).** Lung IHC from a vehicle treated hamster infected with

bioRxiv preprint doi: https://doi.org/10.1101/2022.02.22.481491; this version posted February 24, 2022. The copyright holder for this preprint (which was not certified by peer review) is the author/funder. This article is a US Government work. It is not subject to copyright under 17 USC 105 and is also made available for use under a CC0 license.

Omicron exhibiting frequent immunoreactivity associated with focus of pneumonia (brown color). **(C) Lung H&E (40x).** Lung H&E of a MK-4482 treated hamster infected with Omicron showing normal pathology. **(D) Lung IHC (40x).** Lung IHC of a MK-4482 treated hamster infected with Omicron exhibiting absence of immunoreactivity. Each panel shows a lung section from a representative hamster. Bar = 500 µm
